# Editorial: Insights in *Toxoplasma* Biology and Infection—15th Biennial Meeting on *Toxoplasma* Biology and Toxoplasmosis

**DOI:** 10.3389/fcimb.2021.652637

**Published:** 2021-03-09

**Authors:** Jorge Enrique Gomez-Marin, Alejandra de-la-Torre

**Affiliations:** ^1^ Grupo de Estudio en Parasitología Molecular (GEPAMOL), Centro Investigaciones Biomédicas, Facultad de Ciencias de la Salud, Universidad del Quindío, Armenia, Colombia; ^2^ Escuela de Medicina y Ciencias de la Salud, Grupo NeURos, Centro de Neurociencia (NeuroVitae), Universidad del Rosario, Bogotá, Colombia

**Keywords:** *Toxoplasma gondii*, toxoplasmosis, drug design, *in vivo* model, ocular toxoplasmosis, immune response

## Introduction

**Figure 1:**
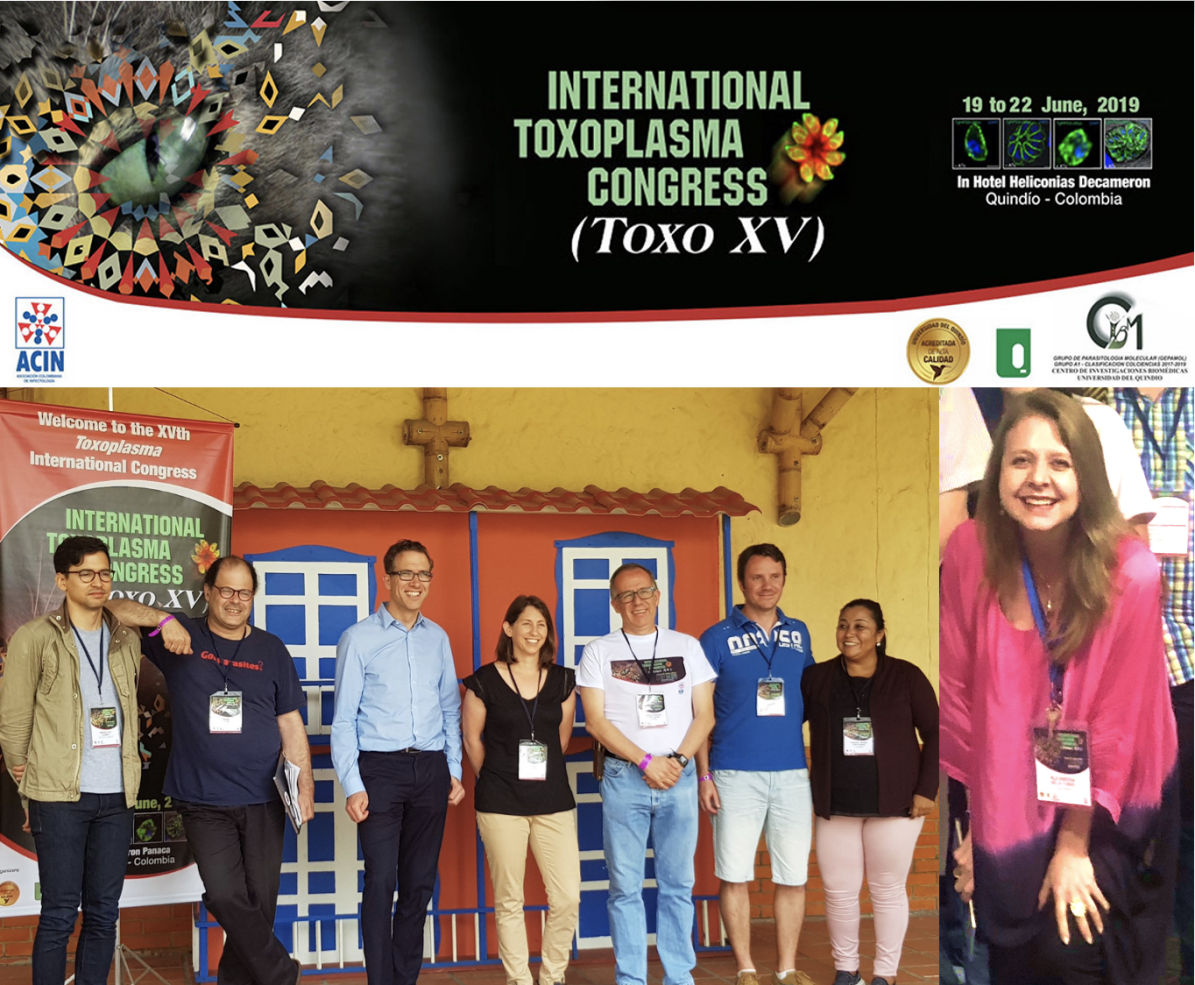
Scientific Committee of the XVth Biennal *Toxoplasma* Biology meeting from left to right: Sebastian Lourido, David Roos, Jeroen Saeij, Karen Shapiro, Jorge Gomez-Marin, Aurelien Dumetre, Fabiana Lora- Suarez and Alejandra de-la-Torre.

The 15th Biennial Meeting on *Toxoplasma* Biology and Toxoplasmosis was held between 19–22 June 2019 in Colombia. The Scientific Committee of this event (**Figure 1**) organized a program that gathered worldwide experts discussing new knowledge advancements regarding the protozoa and the infection caused by it. Numerous topics were addressed, including not only basic parasite-biology mechanisms, but also immune response, diagnostic tools, development of new therapies and public health aspects of *T. gondii* infection. Eleven articles and 118 authors are part of this special Research Topic published in Frontiers in Cellular and Infection Microbiology, which offers a comprehensive view of relevant work presented during the global conference. In this Editorial, we will comment briefly the main aspect addressed for these works and how they improve our current knowledge for this important zoonotic infection.

One basic mechanism of *Toxoplasma* biology is the process by which centromeres are held in position at the nuclear envelope and keep track of the position of their chromosomes, without condensing their chromatin during division. The work published on this Research Topic showed that centromere-associated protein interacts with chromatin and that chromatin binding factors at the centromeres mediate the maintenance of their localization at the periphery of the nucleus (Francia et al.). This data shed light about how Apicomplexa coordinates chromosomes during division. Another basic aspect is how *T. gondii* influences host cell’s biological processes. The work reported by Vieira et al. showed the disruptive effects of this parasite in one murine myoblast cell line, where *T. gondii* established a pro-inflammatory environment extended to neighboring cells and damaged their response to myogenic stimuli. Additional evidence of the modulation of *T. gondii* on inflammatory host signal pathway was provided by the transcriptome analysis by Li et al., which analyzed how *Toxoplasma*-ROP18 virulent factor altered the expression of 750 genes (467 upregulated genes and 283 downregulated genes) in HEK293T cells. This data provided new understanding into how ROP18 may influence these processes by altering the expression of genes, transcription factors and pathways, laying the foundations for future *in vitro* and *in vivo* studies.

Immune response in animal models others than mouse was analyzed by Rahman et al., which showed how pigs can serve as an interesting model for studying the *T. gondii* infection kinetics in the gut. The results suggest that upon ingestion the parasite first enters the host at the duodenum and then disseminates to other tissues. This is associated with the activation of IFN γ secreting immune cells. These findings lay a foundation for further study on the early stages of *T. gondii* intestinal infection and might inform strategies aimed at preventing initial invasion of the host by this parasite (Rahman et al.).

Considering the epidemiological factors of this zoonosis, Blaizot et al. described the investigation of a toxoplasmosis outbreak in a remote Amerindian community. This work highlighted the probable multifactorial origin of this infection, underscoring new life habits among an indigenous population which live in close contact with the Amazon rainforest. Sedentary settlements had been built in the last few decades without providing safe water sources, increasing the risk of parasite circulation in cases of dangerous new habits such as cat domestication. The authors recommended the pursuit of a “One Health” strategy of research involving medical anthropology, veterinary medicine, and public health, for a better understanding of the transmission routes and the presence of this zoonotic disease.

Studies in human cells about the immune response to the parasite modified by parasite virulent proteins ROP18 and ROP16 were reported by Hernandez-de-los-Rios et al. The research group studied infecting cells with knockout parasite for each of these proteins, and how the secretion by peripheral blood polymorphic mononuclear cells of proinflammatory cytokines was influenced by the host’s polymorphisms in the cytokine genes. The findings suggest that the immune response to the parasite in humans does not only depend on the presence of parasite virulence factors like ROP16 and ROP18, but also on the host genetic susceptibility to the infection (Hernández-de-los-Ríos et al.).

One major aspect of human toxoplasmosis is the retinal involvement. An interesting review by de Campos et al. analyzed the available evidence of the effects that congenital TORCH (**T**oxoplasmosis – **O**ther – **R**ubella – **C**ytomegalovirus - **H**erpes) infections may cause to the developing retina and the cellular and molecular aspects of these diseases, with special emphasis on congenital ocular toxoplasmosis. While some questions remain unanswered, this work gives an insight in which key factors are implicated on retinal damage during *in utero* infection, for further research in the pathophysiology of congenital ocular toxoplasmosis (Campos et al.). Again, in relation to ocular toxoplasmosis, Nakashima et al. investigated the correlation of serum IgG anti-*T. gondii* antibody concentrations with Nested PCR. This work was developed considering that the influence of IgG anti-*T. gondii* antibodies in molecular analysis carried out in peripheral blood remain unclear and considering that blood transfusion and organ transplantation represent different forms for *T. gondii* transmission, apart from food and water-borne sources. Results from chronically infected healthy blood donors showed that variations in the serum IgG anti-*T. gondii* antibody concentrations do not correlate to the parasitemia detected by Nested PCR. Similarly, Murata et al. compared serological methods such as ELISA and ELFA, as well as molecular cPCR, Nested PCR and qPCR, for the diagnosis of ocular *T. gondii* infection. The authors showed that the combined use of certain tests along with clinical evaluation and follow up could be useful for the correct diagnosis of *T. gondii *infection (Murata et al.).

A new strategy that is promising for diagnosis is the aptamer assay that uses short, single-stranded oligonucleotides that bind to targets with high affinity and specificity by folding into tertiary structures. The detection of the virulent protein *Toxoplasma* ROP18 protein in human serum with aptamer, signaled that detection of this protein was related with more severe forms of congenital toxoplasmosis suggesting its use as prognostic biomarker (Vargas-Montes et al.).

Finally, one major finding reported in this Research Topic is the development of a powerful tetrahydroquinolone, JAG21, which can eliminate apicomplexan parasites in tissues (McPhillie et al.). This is an extraordinary achievement in *in vivo* models because, until now, there are no drugs that can eliminate tissue cysts of the parasite responsible of reactivations during the host’s period of life. The authors created a next generation lead compound with high *in vitro *and *in vivo* efficacy against *T. gondii* tachyzoites, bradyzoites and established encysted organisms (McPhillie et al.). This compound is promising and deserves further development through preparation of advanced formulations and testing in further studies of pharmacokinetics, efficacy, and safety.

In summary, this Research Topic shows significant advances made in the study of *T. gondii* using *in-vitro*, *in-vivo* and animal models. The results presented here will illuminate the pathway to create an effective clinical response to this public health key issue.

## Author Contributions

JG and AT drafted and edited the editorial. All authors contributed to the article and approved the submitted version.

## Conflict of Interest

The authors declare that the research was conducted in the absence of any commercial or financial relationships that could be construed as a potential conflict of interest.

